# Liver-Resident Memory CD8^+^ T Cells: Possible Roles in Chronic HBV Infection

**DOI:** 10.3390/ijms22010283

**Published:** 2020-12-30

**Authors:** Ji Won Han, Eui-Cheol Shin

**Affiliations:** 1 Laboratory of Immunology and Infectious Diseases, Graduate School of Medical Science and Engineering, Korea Advanced Institute of Science and Technology, Daejeon 34141, Korea; 2 Division of Gastroenterology and Hepatology, Department of Internal Medicine, College of Medicine, The Catholic University of Korea, Seoul St. Mary’s Hospital, Seoul 06591, Korea

**Keywords:** tissue-resident T cell, liver-resident T cell, chronic HBV infection

## Abstract

Achieving a functional cure for chronic hepatitis B virus (HBV) infection or complete elimination of HBV covalently closed circular DNA (cccDNA) has been challenging in the treatment of patients with chronic HBV infection. Although novel antivirals are being investigated, improving HBV-specific adaptive immune responses is also important for durable viral clearance. Tissue-resident memory CD8^+^ T (T_RM_) cells were recently reported as a T-cell population that resides in peripheral tissues and does not recirculate. T_RM_ cells have been studied in the livers of mice and humans. Liver T_RM_ cells have distinct characteristics compared to T cells in peripheral blood or other tissues, which may be associated with the unique microenvironment of the liver. In this review, we describe the characteristics of liver T_RM_ cells and their implications in chronic HBV infection. We emphasize that liver T_RM_ cells can be an immunotherapeutic target for the treatment of chronic HBV infection.

## 1. Introduction

Hepatitis B virus (HBV) is a major risk factor for liver cirrhosis (LC) and hepatocellular carcinoma (HCC). The prevalence of chronic HBV infection is 3.5%, with 257 million infected people worldwide [1]. Although effective antiviral agents have been developed, current antiviral treatments do not eliminate covalently closed circular DNA (cccDNA), a persistent form of the HBV genome, in infected hepatocytes [2]. Furthermore, HBV-specific immune responses are insufficient for elimination of the virus in chronic HBV infection [3]. For effective control of HBV, collaboration of the innate and adaptive immune responses is crucial [4]. CD8^+^ cytotoxic T cells are one of the major players in the adaptive immune system, which specifically recognizes viral epitopes loaded on major histocompatibility complex (MHC) class I molecules, thereby eliminating viruses by killing virus-infected cells or releasing antiviral cytokines [5]. The International Coalition to Eliminate HBV (ICE-HBV) has suggested that future research on HBV immune control is one of the important aims to achieve a cure for chronic hepatitis B [2]. However, studies that have tried to restore HBV-specific immune responses have not been successful. In ex vivo experiments, blocking inhibitory receptors had only partial effects on the restoration of HBV-specific T-cell [6] and B-cell [7] responses. A recent pilot human trial showed that blocking programmed cell death-1 (PD-1) had a partial effect in decreasing the level of HBV surface antigen (HBsAg) [8].

Tissue-resident memory T (T_RM_) cells were recently identified as a non-circulating T-cell population that performs frontline antiviral defense in various peripheral organs, including the liver [9,10,11]. T_RM_ cells do not egress to the blood circulation, but reside in peripheral tissues [9,10,11]. The liver is an immunologically unique organ, with tolerogenic features associated with continuous exposure to gut-derived food-related antigens or microbial products [12]. Therefore, the characteristics of liver T_RM_ cells may be distinct compared to T_RM_ cells in other organs, and understanding them may be helpful to controlling chronic HBV infection. Recently, several reports have been published regarding liver T_RM_ cells in mice and humans. In this review, we describe recent studies of liver T_RM_ cells and discuss whether they could be potential therapeutic targets for the treatment of chronic HBV infection.

## 2. General Characteristics of T_RM_ Cells

T_RM_ cells refer to a T-cell population that resides in peripheral organs, does not recirculate to the blood, and responds rapidly and robustly to local antigenic stimulation. They are now considered a memory T-cell subset [9], though they generally exhibit a phenotype of effector memory T (T_EM_) cells in terms of CCR7 and CD45RA expression [9,10,11]. Therefore, this population broadly includes CD4^+^ T cells, FoxP3^+^ regulatory T cells, and innate-like T cells, such as γδ T cells, natural killer T cells, and mucosal-associated invariant T (MAIT) cells. However, most studies of T_RM_ cells have defined them as a CD8^+^ T-cell population with the typical phenotypes of tissue residency. Therefore, this review mainly focuses on the CD8^+^ T-cell population.

**Figure 1:**
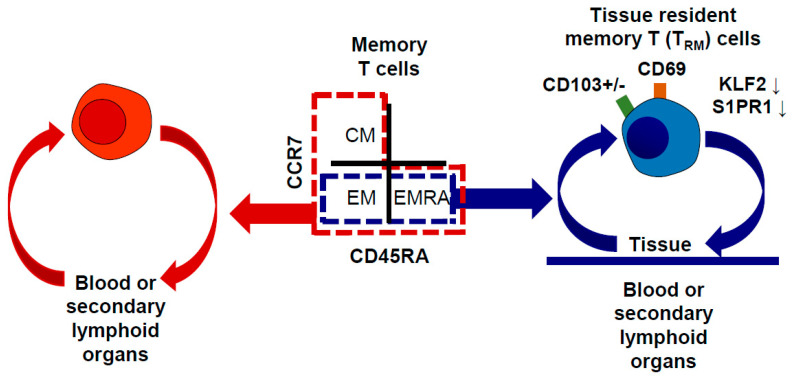
Characteristics of tissue-resident memory T (T_RM_) cells. T_RM_ cells express CD69 and CD103, though CD103 expression is variable depending on the type of peripheral organ. These cells also downregulate *KLF2* and *S1PR1* and cannot egress to the blood or secondary lymphoid organs; therefore, they reside in the peripheral tissues. T_RM_ cells do not express CCR7 and exhibit an effector memory T cell (T_EM_) phenotype or effector memory T cells re-expressing CD45RA (T_EMRA_) phenotype. However, circulating memory T cells also have central memory T (T_CM_) cells that express CCR7 but not CD45RA. CM, central memory; EM, effector memory; EMRA, effector memory re-expressing CD45RA.

T_RM_ cells were first described in mice using parabiosis [13,14,15] and intravascular staining [15,16]. Figure 1 shows the representative characteristics of T_RM_ cells compared to the circulating T-cell population. They generally express CD69 but do not express *S1PR1* and *KLF2*, which prevent T cells from egressing out of the peripheral organ [9,10,11]. T_RM_ cells also downregulate molecules associated with homing to the lymph nodes, such as CD62L and CCR7 [9,10,11]. Consequently, they can constantly reside in the peripheral tissues and respond to secondary antigen stimulation, thereby functioning as a frontline protector against infection. In addition to CD69, the integrin molecule CD103 has been considered a canonical marker of T_RM_ cells, but some mouse studies have reported that T_RM_ cells in the liver [17,18] and kidney [19] do not express CD103. PD-1, CD49a, CD101, and CXCR6 are also known to be upregulated in T_RM_ cells, though their expression levels are different according to the type of tissue. Cytokines, such as tumor growth factor-β (TGF-β) and interleukin (IL)-15, play a role in the development of T_RM_ cells [20]. *Hobit* and *Blimp1* are important transcriptional regulators for the function and maintenance of T_RM_ cells [17], and the aryl hydrocarbon receptor and Notch signaling are associated with T_RM_ maintenance [21,22]. Upon antigenic stimulation, such as infection or tumor growth, T_RM_ cells respond rapidly by proliferating, secreting cytokines such as interferon-γ (IFN-γ), tumor necrosis factor (TNF), and IL-2, and exerting cytotoxicity. Moreover, they trigger adaptive and innate immune responses, such as dendritic cell (DC) maturation, NK cell activation, and B cell recruitment [23]. Therefore, murine studies have revealed that T_RM_ cells have unique phenotypes and transcriptional programs that are associated with their local maintenance and function.

Fewer studies have been performed on T_RM_ cells in human tissues due to the difficulties obtaining tissue samples compared to mice and difficulties proving true persistence within peripheral tissues. However, evidence from human studies is accumulating. In human T_RM_ studies, CD69 and CD103 have been used to define T_RM_ cells in various peripheral organs, and phenotypically defined human T_RM_ cells share core characteristics of mouse T_RM_ cells. Importantly, human studies of T_RM_ cells have revealed associations with disease activity in infection, cancer, autoimmune diseases, and transplantation [20].

## 3. Liver T_RM_ Cells

### 3.1. General Features of Liver T Cells

Before the concept of T_RM_ cells was established, reports described the characteristics of liver T cells, focusing on the mechanism of trapping, activation, and tolerance. An old report briefly but comprehensively showed the characteristics of hepatic T-cell responses [24]. Activated T cells were trapped in the liver but then underwent apoptosis, suggesting that the liver accumulates T cells but also induces their tolerance [24].

The retention of circulating T cells within liver sinusoids is first induced by docking to platelets, which can attach to sinusoidal hyaluronan in a CD44-dependent manner, and then the T cells crawl along the liver sinusoids during hepatocellular antigen recognition [25]. Another report demonstrated that the trapping of T cells within liver sinusoids may occur via liver sinusoidal epithelial cells (LSECs), Kupffer cells, and hepatic stellate cells (HSCs), which upregulate adhesion molecules such as ICAM-1, VCAM-1, and VAP-1 [26]. Thus, liver T-cell trapping and crawling within the sinusoids may allow communication with other cell populations within the liver.

Under stable conditions, numerous gut-derived materials enter the liver via the portal vein. Therefore, liver T cells are instructed by cells in the hepatic microenvironment to be tolerant. HSCs can restrict hepatic T-cell responses via their enhanced expression of programmed death-ligand 1 (PD-L1), which induces T-cell apoptosis [27]. Furthermore, mouse HSCs can interfere with CD8^+^ T cells in an ICAM-1-dependent manner and inhibit their activation by antigen-presenting cells, leading to apoptosis [26]. They also contribute to the induction of regulatory T (Treg) cell development by retinoic acid and TGF-β secretion [28]. Kupffer cells can expand IL-10-producing antigen-specific Treg cells [29] and inhibit DC-induced antigen-specific T-cell activation [30], and this suppression of T-cell responses may be associated with the surface expression of PD-L1 [29]. LSECs induce CD4^+^ T cells to differentiate to Treg cells in an IL-10- and PD-1-dependent manner [31,32]. Furthermore, antigen presentation on LSECs can induce antigen-specific T-cell tolerance [33] via the PD-1/PD-L1 interaction [34]. Hepatocytes can prime CD8^+^ T cells but induce BIM-dependent clonal T-cell deletion [35]. Taken together, these features of liver T cells induced by communication with other cells within the liver may be associated with the tolerant characteristics of liver T cells.

### 3.2. Mouse Liver T_RM_ Cells

The term “liver-resident memory T cell” was first used in a murine study that performed a microarray analysis to identify the unique transcriptional profile of liver CD8^+^ T cells induced by malarial immunization [36]. This study revealed distinct transcriptional profiles of liver T cells compared to the splenic CD8^+^ T cells, including downregulation of *KLF2*, *S1PR1*, and *CD62L*, as well as upregulation of *CD69* [36]. Another study by the same group revealed that CXCR6 is important for shaping and maintaining hepatic memory CD8^+^ T cells [37].

Mouse T_RM_ cells have been shown to upregulate CD69, and they have common transcriptional signatures, such as *Hobit* and *Blimp1,* in all types of peripheral tissues, including liver T_RM_ cells [17]. This report showed that liver T_RM_ cells also share a transcriptional signature with other T_RM_ cells, but do not express CD103, which is commonly expressed by T_RM_ cells from other tissues [17].

A recent report by Fernandez-Ruiz et al. comprehensively showed the characteristics of mouse liver T_RM_ cells [18]. CD69^+^ liver T_RM_ cells could be induced by malarial immunization, persist in the liver sinusoids, and patrol the liver sinusoids [18]. Another recent report revealed that upregulation of LFA-1 is responsible for the patrol and persistence of liver T_RM_ cells within liver sinusoids [38]. Furthermore, liver T_RM_ cells were essential for the protective immune responses following malarial immunization via production of cytokines such as IFN-γ and TNF, and the expression of cytotoxic markers such as CD107a and granzyme B, which was proven by depletion of liver T_RM_ cells by targeting CXCR3 [18]. Phenotypically, liver T_RM_ cells highly expressed CXCR6, CXCR3, and CD101 but did not express CD62L and KLRG1 [18]. In addition, this study confirmed that mouse liver T_RM_ cells do not express CD103 [18]. The lack of CD103 expression in liver T_RM_ cells, unlike T_RM_ cells from other tissues, may be due to their location in the liver sinusoids, which is continuous with the blood stream. Furthermore, this characteristic may be associated with the unique induction mechanism of liver T_RM_ cells compared to other T_RM_ cells; they can be induced by vaccination or infection outside the epithelial tissues [18,39]. This was proven by showing that adoptive transfer of in vitro activated T cells results in liver T_RM_ cells [39], which is reminiscent of the old report that activated T cells can be trapped within the liver [24]. Taken together, liver T_RM_ cells share core characteristics of the T_RM_-cell population, but have distinct phenotypic characteristics that may be related to their induction mechanism and location.

### 3.3. Human Liver CD69^+^CD8^+^ T Cells

Stelma et al. briefly characterized human liver CD69^+^CD8^+^ T cells that have tissue-resident phenotypes for the first time using liver tissue samples [40]. They observed that >50% of liver CD8^+^ T cells expressed CD69, and the CD69^+^ subpopulation downregulated *S1PR1* and *KLF2* compared to the CD69^−^ subpopulation [40]. These cells also overexpressed CXCR6 and PD-1 and exhibited memory (CD45RA^−^CD27^+^) or effector memory (CD45RA^±^CD27^−^) phenotypes. These phenotypic characteristics are consistent with those of mouse liver T_RM_ cells. However, this report observed different characteristics than those of mouse liver T_RM_ cells [40]. First, an average of 12.4% of human liver CD69^+^CD8^+^ T cells expressed CD103, whereas the mouse liver CD69^+^ T_RM_ cells do not express CD103 [17,18]. Second, although a previous murine study showed that liver T_RM_ cells express more granzyme B, CD107a, IFN-γ, and TNF upon stimulation than non-T_RM_ liver T_EM_ cells [18], this human study showed reduced expression of granzyme B and perforin by liver CD69^+^CD8^+^ T cells, showing a hypofunctional cytotoxic capacity [40]. As next steps, we and others attempted to characterize human liver CD69^+^CD8^+^ T cells, dividing them into CD103^+^ and CD103^−^ subpopulations.

#### 3.3.1. Human Liver CD69^+^CD103^+^CD8^+^ T_RM_ Cells

Pallet et al. described the characteristics of human liver CD69^+^CD103^+^CD8^+^ T cells (CD103^+^ subpopulation) in healthy donors and patients with chronic HBV infection using samples from liver biopsies, perfusates obtained during liver transplantation (LT), and tissues obtained during surgery for liver metastases of colorectal cancer [41]. This study investigated the CD103^+^ subpopulation compared to blood CD8^+^ T cells and liver CD69^−^CD103^−^CD8^+^ T cells, and found that the CD103^+^ subpopulation comprised ~10% of memory CD8^+^ T cells within the healthy liver [41].

Following confirmation of their location within the liver sinusoids by immunofluorescence, Pallet et al. found that CXCR6 was highly expressed in the CD103^+^ subpopulation [41]. LSECs, Kupffer cells, and hepatocytes express CXCL16, which is a ligand for CXCR6 and plays a role in the adhesion, accumulation, and maintenance of intrahepatic T cells [42,43,44]. Therefore, their observation confirmed that CXCR6 expression may also be an important hallmark of the human liver T_RM_-cell population. Furthermore, this report showed that the CD103^+^ subpopulation has a unique transcriptional signature, T-bet^lo^Eomes^lo^Blimp-1^lo^Hobit^lo^ [41]. Hobit expression had a converse pattern compared to mouse liver T_RM_ cells [17], suggesting that human liver T_RM_ cells have distinct characteristics from mouse liver T_RM_ cells and emphasizing the need for detailed characterization of human liver T_RM_ cells.

Consistent with the T_RM_ cells in other tissues, human liver CD69^+^CD103^+^CD8^+^ T_RM_ cells highly express PD-1 [41]. As noted above, LSECs and hepatocytes express PD-L1, which can interact with PD-1, thereby inhibiting the function of T cells. Although the role of PD-1 expression in liver T_RM_ cells needs to be elucidated, it may be associated with T-cell-induced liver injury. This is supported by a recent report that PD-L1 expression of liver-resident NK cells attenuates liver T-cell-induced liver injury [45]. However, regardless of PD-1 expression, they found similar production of IFN-γ and superior production of IL-2 by the CD103^+^ subpopulation compared to blood CD8^+^ T cells and liver CD69^−^CD8^+^ T cells in functional analyses using in vitro stimulation [41]. In ex vivo analyses, the CD103^+^ subpopulation expressed more perforin than the other subpopulation [41], suggesting that they were ready to respond to local antigenic simulation and perform cytotoxic functions. Pallet et al. thoroughly analyzed the characteristics of the human liver CD69^+^CD103^+^CD8^+^ T_RM_ cells, but the characteristics of human liver CD69^+^CD103^−^CD8^+^ T cells—which comprise most of the human liver CD69^+^CD8^+^ T cells—were not addressed.

#### 3.3.2. Human Liver CD69^+^CD103^−^CD8^+^ T_RM_-Like Cells

Recently, our group reported the characteristics of human liver CD69^+^CD103^−^CD8^+^ cells (CD103^−^ subpopulation) using liver tissues and perfusates from healthy donors and LT recipients [46]. Table 1 compares the CD103^+^ and CD103^−^ subpopulations in terms of transcription factors, protein expression, function, survival, and antigen specificity. We investigated the phenotypes of the CD103^−^ subpopulation and found similar expression of CXCR6 as the CD103^+^ counterpart, in addition to a similar lack of expression of *S1PR1* and *KLF2*, suggesting that the CD103^−^ subpopulation also has tissue-resident phenotypes [46]. Distinctively, LFA-1 was significantly upregulated in the CD103^−^ subpopulation, which is consistent with findings in mouse liver T_RM_ cells [38]. Using immunofluorescence, we confirmed that they were also located in the liver sinusoids [46]. These findings indicate that the CD103^−^ subpopulation has phenotypic characteristics of liver T_RM_ cells. However, they had intermediate expression of CD49a and high expression of Eomes compared to their CD103^+^ counterparts; therefore, whether this population is a bona fide T_RM_ cell population needs to be elucidated [46]. For this reason, Swadling et al. referred to the CD103^−^ subpopulation as human liver CD69^+^CD103^−^CD8^+^ “T_RM_-like” cells [47].

In subsequent functional analyses, the CD103^−^ subpopulation had less cytokine productive capacity compared to the CD103^+^ subpopulation upon anti-CD3 stimulation [46], which is in line with the tolerant feature of hepatic immune responses. The CD103^−^ subpopulation highly expressed PD-1, which is similar to their CD103^+^ counterparts [46], suggesting that these cells may be affected by PD-L1-expressing cells within the liver. It may be of interest to investigate whether targeting the PD-1/PD-L1 axis can improve the function of human liver T_RM_ cells, including the CD103^−^ subpopulation. However, the CD103^−^ subpopulation was the major functioning T-cell population in the liver in terms of numbers, although they are hypofunctional on a per-cell basis. Furthermore, the CD103^−^ subpopulation was less susceptible to activation-induced cell death than the CD103^+^ subpopulation, and presented a terminally differentiated phenotype and shorter telomere length [46], suggesting that it may be a persisting population over the long term. Apparently, they may play a role as an immunological sentinel of the liver in terms of their overall functionality and sustainability.

Interestingly, we found that CD8^+^ T cells specific for non-hepatotropic viruses such as cytomegalovirus (CMV), herpes simplex virus (HSV), and Epstein–Barr virus (EBV) were present in the CD103^−^ subpopulation but not the CD103^+^ subpopulation [46]. Although the mechanism is unclear, it may be linked to older studies showing that mouse liver T_RM_ cells can be induced from activated T cells by antigens that are not located within the liver as in the adoptive transfer experiment noted above [18,39]. These findings suggest that the CD103^−^ subpopulation consists of T cells of heterogeneous origin and may contribute to bystander T-cell activation and the immunopathogenesis of liver diseases. We recently showed that bystander-activated CD8^+^ T cells are associated with liver damage in acute hepatitis A via IL-15 [48]. Therefore, we investigated whether this major intrahepatic T-cell population is activated by IL-15, finding that the IL-15-stimulated CD103^−^ subpopulation can exert cytotoxicity [46]. These findings of bystander activation suggest that the CD103^−^ subpopulation can act as a double-edged sword in liver immunity.

Considering the unique environment of the liver, we hypothesized that human liver CD69^+^CD103^−^CD8^+^ T_RM_-like cells may also be regulated by a distinct transcriptional regulator. In the transcriptome analyses, we found that hypoxia-induced factor-2α (HIF-2α) was upregulated in the CD103^−^ subpopulation compared to the CD103^+^ subpopulation, and its expression was associated with the function and survival of the CD103^−^ subpopulation [46]. It is possible that the liver also has an hypoxic microenvironment [49], and it might be associated with the HIF-2α upregulation in the CD103^−^ subpopulation. However, HIF-1α, which is also upregulated by hypoxia, was not upregulated in the CD103^−^ subpopulation, suggesting that there may be a distinct mechanism underlying HIF-2α induction [46]. In addition, although the role of HIF-1α in effector T-cell responses has been reported previously [50,51], the role of HIF-2α is still unclear. Intriguingly, ex vivo HIF-2α inhibition reduces function and survival, specifically in the CD103^−^ subpopulation [46].

## 4. Liver T_RM_ Cells in Chronic HBV Infection

T-cell responses play a crucial role in the clinical outcome of chronic HBV infection [52]. However, with chronic antigenic stimulation, HBV-specific T cells are functionally exhausted, and inhibitory molecules such as PD-1, T-cell immunoglobulin and mucin-domain containing-3 (TIM-3), and cytotoxic T-lymphocyte-associated protein-4 (CTLA-4) are upregulated [53,54]. Serum HBsAg clearance in mice [55] and decreased viral load by nucleoside analogue (NUC) treatment [56] had a limited effect on the HBV-specific T-cell responses. In addition, in vivo treatment of anti-PD-1 in patients with chronic HBV infection resulted in a small degree of HBsAg reduction, and only 1 of 12 patients achieved HBsAg seroconversion [8], suggesting that additional immunotherapeutic strategies are needed to improve HBV-specific T-cell responses.

Importantly, studies seeking immunological targets for the treatment of chronic HBV infection beyond the traditional immune-checkpoint inhibitors are actively under investigation. Among them, one study reported that CXCL13-mediated intrahepatic CXCR5^+^CD8^+^ T-cell accumulation was correlated with a decrease in HBsAg level in a HBV mouse model [57]. In addition, a recent report using the HBV mouse model revealed that hepatic priming of intrahepatic CD8^+^ T cells induced dysfunctional CD8^+^ T-cell responses, which could be restored by IL-2 treatment but not by anti-PD-L1 blockade [58]. These findings suggest liver T cells as a possible treatment target for chronic HBV infection.

The characteristics of HBV-specific CD8^+^ T_RM_ cells in humans were reported recently. Pallet et al. [41] first investigated the characteristics of human liver HBV-specific CD8^+^ T cells and found that the CD103^+^ subpopulation was enriched in patients with chronic infection. More than 80% of HBV-specific CD8^+^ T cells in the liver mostly expressed CD69, and the proportion was similar for the CD103^+^ and CD103^−^ subpopulations [41]. Importantly, the frequency of the CD103^+^ subpopulation inversely correlated with the HBV viral load [41], suggesting that this subpopulation may play a role in the control of HBV. Compared to blood CD8^+^ T cells, liver CD69^−^CD8^+^ T cells, or the CD103^−^ subpopulation, the CD103^+^ subpopulation produced high levels of IL-2 upon HBV-peptide stimulation [41], which may further enhance HBV-specific T-cell responses [58]. Furthermore, the CD103^+^ subpopulation in patients with chronic HBV infection was upregulated PD-1 compared to healthy controls [41]. Thus, this study emphasized that human liver CD69^+^CD103^+^ T_RM_ cells are a promising target for the treatment of chronic HBV infection [59].

Our group also recently reported the characteristics of human liver HBV-specific CD8^+^ T cells, focusing on the CD69^+^CD103^−^CD8^+^ T_RM_-like cells [46]. Consistent with the study by Pallet et al., the CD103^−^ and CD103^+^ subpopulations had similar frequencies within liver HBV-specific CD8^+^ T cells [46]. Importantly, upon HBV-peptide stimulation, the CD103^−^ subpopulation poorly produced cytokines such as IFN-γ, TNF, and IL-2, on a per-cell basis [46], though they were the major population producing cytokines in terms of numbers. Thus, an investigation into the mechanism underlying its hypofunction is of interest. It is also important to question whether enhancing the function of HBV-specific CD69^+^CD103^−^CD8^+^ T_RM_-like cells can control HBV in patients with chronic HBV infection.

Although we focused on the possible protective roles of liver T_RM_ cells in chronic HBV infection, their pathological features during HBV infection should also be considered. For example, bystander activation of CD8^+^ T cells is associated with the liver damage in acute HAV infection [48], and liver T_RM_ cells comprise non-hepatotropic virus-specific cells and can be activated and function via IL-15 stimulation [46]. Therefore, liver T_RM_ cells might also be associated with the liver damage in HBV infection via bystander activation. Furthermore, our recent study also implicated that the activation of liver CD69^+^CD103^−^ T_RM_-like cells were correlated with the impairment of liver function of LC patients [46]. As lung T_RM_ cells induced age-associated chronic lung fibrotic sequelae after viral pneumonia in a mouse experimental study [60], whether liver T_RM_ cells are linked to the liver fibrosis or cirrhosis in chronic HBV infection should also be proved mechanistically. Finally, accumulation of tumor-localizing T_RM_ cells predicted survival of patients better than the frequency of total CD8^+^ T cells in melanoma [61] and breast cancer [62], although there is limited data available from HCC patients. Because most HCCs are developed from the background LC or liver fibrosis in chronic HBV infection, whether liver T_RM_ cells might be protective or not will be of great importance for the future research.

## 5. Conclusions

Current NUCs effectively suppress HBV replication. Nevertheless, after successful NUC treatment, cccDNA persists in infected hepatocytes. Currently, novel targeted antivirals such as HBV entry inhibitors, nucleocapsid assembly modulators, RNA interference agents, HBsAg release inhibitors, and cccDNA inhibitors are being investigated [60]. Immunomodulatory treatments, including interferons, Toll-like receptor agonists, therapeutic vaccines, and immune-checkpoint inhibitors, are also important to achieve durable viral clearance and a functional cure [60]. Immunomodulatory strategies are supported by a recent in vitro study showing that cccDNA can be reduced by IFN-γ and TNF produced by T cells [61].

The recent studies discussed in this review suggest that HBV-specific liver T_RM_ cells have unique characteristics, and other strategies in addition to immune-checkpoint inhibitors may be needed to improve their function. Furthermore, we need to consider not only the CD8^+^ T_RM_ cells discussed in the present review, but also other liver-resident immune cells, including CD4^+^ T_RM_ cells and tissue-resident B cells, to understand the intrahepatic HBV-specific immune responses and to develop a cure for chronic HBV infection.

## Figures and Tables

**Table 1:** Comparison of the CD103^+^ and CD103^−^ subpopulations among human liver-resident CD69^+^CD8^+^ T cells.

	CD103^+^ T_RM_	CD103^−^ T_RM_-like
Frequency among CD69^+^ cells	~5%	~95%
PD-1	++	++
HIF-2α	+	+++
**Tissue residency**		
***S1PR1***	−	−
***KLF2***	−	−
**CXCR6**	+++	+++
**LFA-1**	++	+++
**CD49a**	++	+
*RUNX3*	+++	−
Memory		
CCR7	−	−
CD45RA	−	++
Terminal differentiation		
CD57	+	++
KLRG1	−	++
Eomes	+	+++
Telomere length	++	+
Activation		
CD38	+	++
HLA-DR	+	+
TCR-dependent function, per cell basis		
Cytokine	++	+
Cytotoxicity	++	+
TCR-dependent function, overall		
Cytokine	+	+++
Cytotoxicity	+	+++
TCR-independent function, per cell basis		
Proliferation	++	+
Cytotoxicity	++	+
TCR-independent function, overall		
Cytotoxicity	+	++
Survival		
Activation-induced cell death	++	+
FAS	++	+
Antigen specificity		
HBV	+	+
IAV	−	+
RSV	−	+
CMV	−	+
EBV	−	+

CMV, cytomegalovirus; EBV, Epstein–Barr virus; HBV, hepatitis B virus; HIF-2α, hypoxia-induced factor-2 alpha; IAV, influenza A virus; PD-1, programmed cell death-1; RSV, respiratory syncytial virus; TCR, T-cell receptor; T_RM_, tissue-resident memory T cell. Relative expression or frequency is presented from minimal − to highest +++ for each marker among the CD103^+^ and CD103^−^ subpopulations.
